# Peripheral retinal leakage in POEMS syndrome

**DOI:** 10.1186/s40942-020-00278-1

**Published:** 2021-01-12

**Authors:** Andrew Rising Carey, Praveen Jeyaseelan

**Affiliations:** 1 Neuro-Ophthalmology, Wilmer Eye Institute, 600 N Wolfe Street, Baltimore, MD 21287 USA; 2 Clinical Neuro-Ophthalmology Fellow, Wilmer Eye Institute, 600 N Wolfe Street, Baltimore, MD 21287 USA

**Keywords:** POEMS syndrome, Disc leakage, Peripheral retinal leakage, Fundus fluorescein imaging, VEGF

## Abstract

**Background:**

POEMS (polyneuropathy, organomegaly, endocrinopathy, myeloma protein, skin changes) syndrome is a rare blood disorder with multi-system involvement. The cause is unknown. It is marked by elevated plasma cells, platelets, & VEGF (vascular endothelial growth factor) levels. 52% of patients develop optic disc edema which may be vision threatening but the exact etiology of optic disc edema is uncertain. We report a rare finding of peripheral retinal leakage in POEMS syndrome.

**Case presentation:**

A 60 year-old female with POEMS syndrome presented with bilateral blurred vision. Fundi showed grade 3 disc edema OU. Lumbar puncture showed normal opening pressure. CSF analysis showed elevated proteins with no cells. MRI brain and MR Venogram head were unremarkable. Wide field fluorescein angiography demonstrated multifocal tiny vascular leakage and significant anterior temporal leakage.

**Conclusion:**

The authors hypothesize the disc edema in POEMS syndrome may be caused by increased vascular permeability at the optic disc secondary to increased VEGF (vascular endothelial growth factor) levels. Though disc leakage is a well-documented finding in fundus fluorescein imaging, peripheral retinal leakage in POEMS syndrome is not reported.

## Case report

**Fig. 1:**
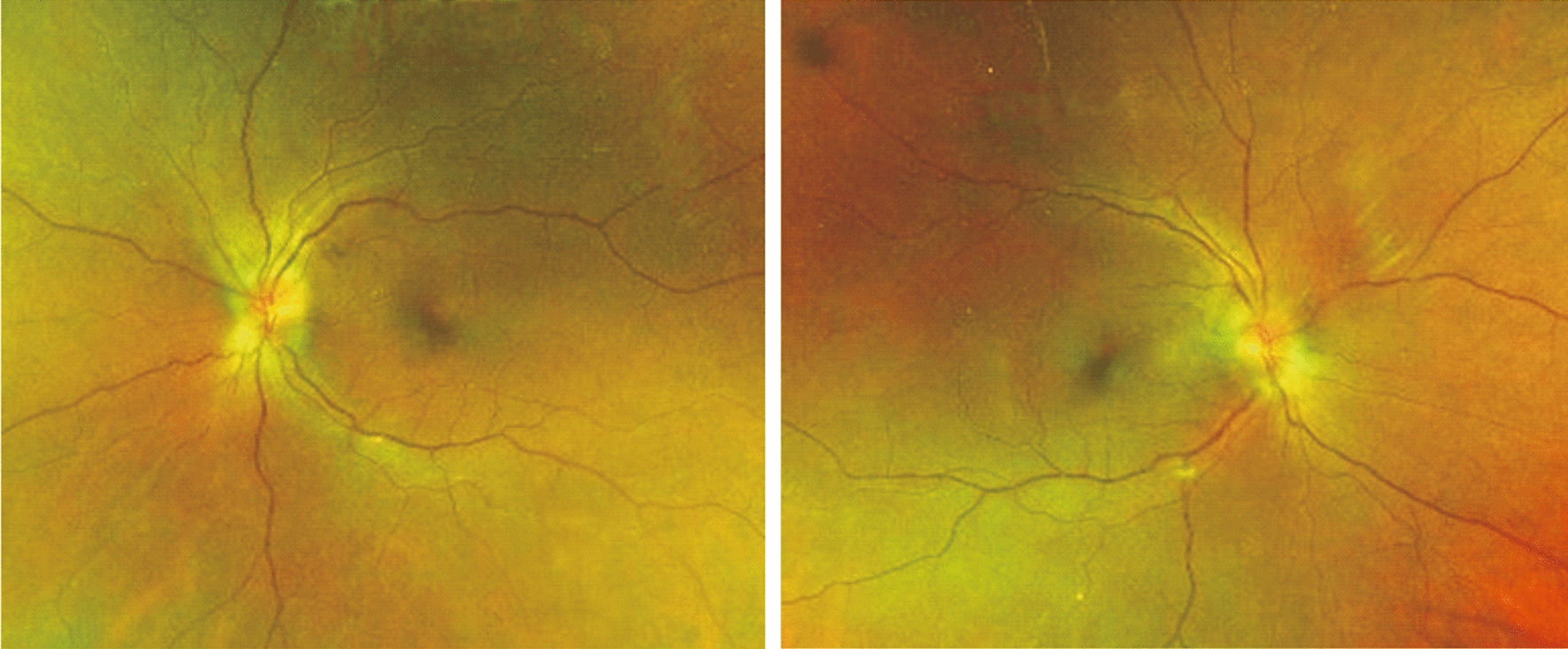
Bilateral optos pseudo-color fundus photography showing disc edema

**Fig. 2:**
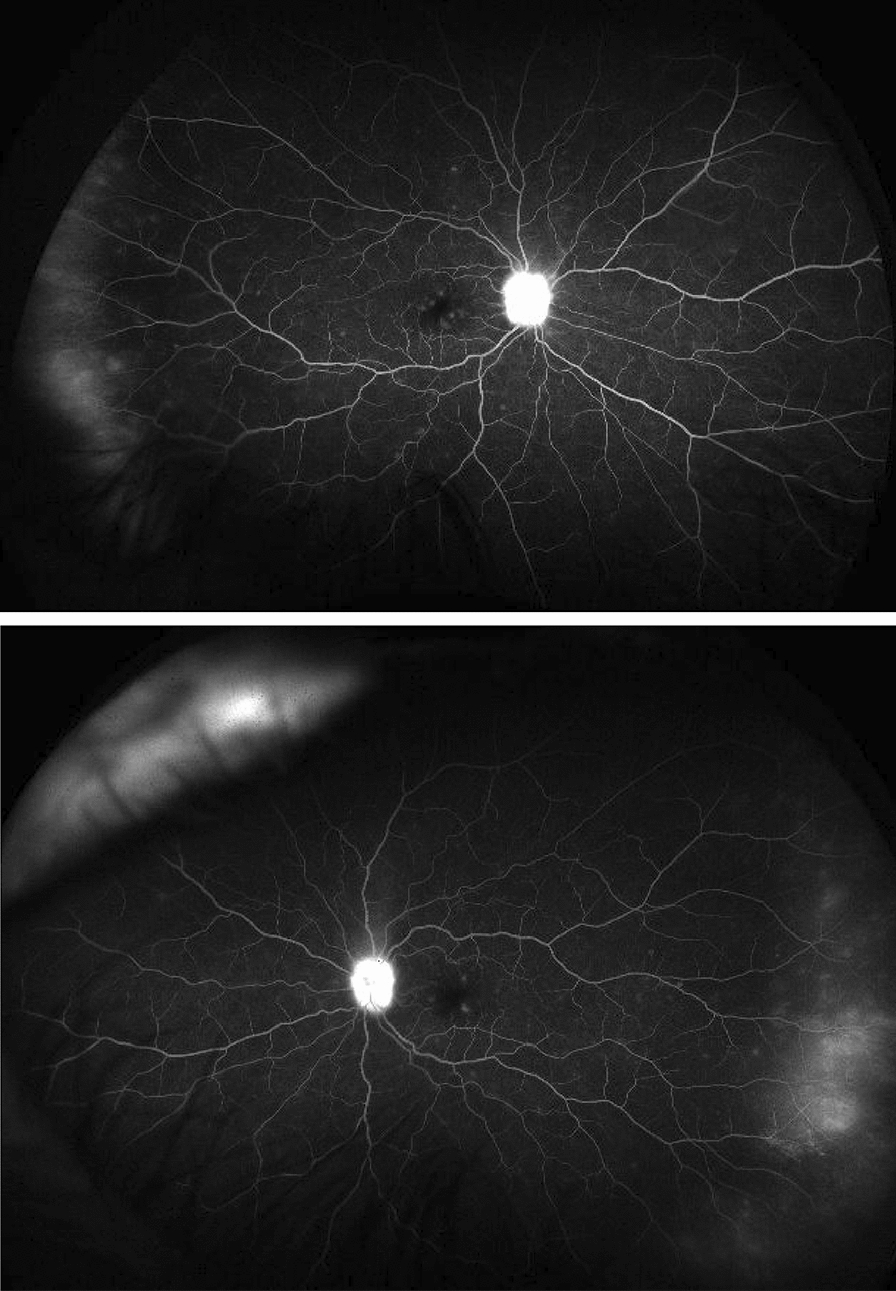
Bilateral optos fluorescein angiography: bilateral disc staining and leakage, multifocal retinal trace leakage, temporal peripheral vascular leakage

A 60 year-old female presented with complaints of bilateral blurred vision associated with occasional floaters and flashes. Two months prior to presentation she underwent uncomplicated, consecutive, cataract surgery with intraocular lens implant. Her medical history was remarkable for slowly progressive weakness of both legs, numbness, difficulty in climbing stairs, swelling of both ankles, and hyper pigmentation over thighs. On examination, her visual acuity was 20/20 with full color vision and normal confrontation fields. There was no relative afferent papillary defect. Fundi showed grade 3 disc edema with dull foveal reflex bilaterally (Fig. 1). OCT (optical coherence tomography) showed RNFL (retinal nerve fiber layer) thickening with normal macular GCL (ganglion cell layer). Fundus fluorescein angiography showed mild macular leakage with multifocal scattered peripheral leakage bilaterally and anterior temporal leakage (Fig. 2). Cranial nerves were normal however she had bilateral 3/5 weakness in lower limbs with negative Achilles reflexes and normal plantar flexor responses (negative Babinski). Sensory system examination showed severely reduced vibration in bilateral toes and fingers. Gait demonstrated wide based sensory gait with no foot drop.

Blood work up showed mild anemia, thrombocytosis (677,000/mm3) and (normal 150,000–400,000/mm3). Bone marrow biopsy showed 10% to 15% kappa-predominant plasma cells. Her M spike was 0.4 g/dl, Serum Immunofluorescence showed positive IgA lambda band. Her total protein, VEGF, IgG, IgA were elevated. Urine immunofixation showed two IgA lambda bands and one free lambda band. Lumbar puncture showed normal opening pressure (13 cm H_2_O) (normal range 10–20 cm H_2_O). CSF analysis showed mildly elevated protein at 62 mg/dl (normal 15–45 mg/dl) with no cells. PET images showed splenomegaly without metabolic evidence of lymphadenopathy or malignancy. MRI brain and MRV did not show any signs of elevated intracranial pressure or dural venous sinus thrombosis/stenosis.

## Discussion

Clinical signs of POEMS that are included in the diagnostic criteria are shown in Table 1. It is a chronic multi systemic disorder with overproduction of pro-inflammatory cytokines like VEGF which causes increased vascular permeability and edema [1–3]. Serum VEGF levels correlates with disease activity, however, VEGF inhibition with systemic bevacizumab failed to result in an effective treatment suggesting VEGF could just be part of a complex cytokine network [2]. Polyneuropathy is attributed to axonal degeneration, uncompacted myelin lamella, and endoneural edema [3].

**Table 1:** Diagnostic criteria for POEMS syndrome: [1]

Requires two mandatory criteria PLUS ≥ 1 major AND ≥ 1 minor criteria
Mandatory criteria
Polyneuropathy (typically demyelinating)
Monoclonal plasma cell-proliferative disorder (almost always λ)
Major criteria
Castleman disease*(lymphoproliferative disorder)*
Sclerotic bone lesions
Vascular endothelial growth factor (VEGF) elevation
Minor criteria
Organomegaly (splenomegaly, hepatomegaly, or lymphadenopathy)
Extravascular volume overload (edema, pleural effusion, or ascites)
Endocrinopathy (adrenal, pituitary, gonadal, parathyroid, thyroid and pancreatic)
Skin changes (hyperpigmentation, hypertrichosis, glomeruloid hemangiomata, plethora, acrocyanosis, flushing, and white nails)
Papilledema
Thrombocytosis/polycythemia

In a retrospective evaluations of 170 POEMS cases, 84% of patients documented at least one endocrine abnormality at presentation [1]. There is a higher incidence of disc edema among patients with IgG Heavy Chain 14q32 translocation [4]. A study done by Bolling et al. showed optic disc edema in 52% of the patients [5]. Optic disc edema was the presenting manifestation of POEMS in 1 case [6]. Among patients with disc edema, 75% had enlarged blind spots bilaterally, others had generalized constriction and bilateral inferior nasal steps. However our patient had normal fields.

Other reported ocular manifestations are macular edema, serous macular detachment, infiltrative orbitopathy, venous sinus thrombosis, uveitis, neovascularization of the disc and peripapillary choroidal neovascularization [5–7]. Elevated intracranial pressure, vasculitis, infiltration of the nerve or increased VEGF levels may contribute to the disc edema. Most patients have normal opening pressure as ICP fluctuations are common in POEMS syndrome, however they can have raised intracranial pressure due to increased CSF proteins. It is believed that the disc edema is not true papilledema and its most related to optic disc vasculitis [2, 5]. Disc edema tends to persist even after the treatment with monoclonal antibodies, however the tendency to develop optic atrophy is rare [5]. There was a statistically significant difference in VEGF levels among patients with and without disc edema. Literature shows no statistical difference in mean lumbar puncture opening pressure [7]. Body mass index had no statistical association with disc edema in POEMS [7].

In the vast majority of cases, even after treatment with steroids and acetazolamide, the macular edema disappeared but the disc edema persisted for a longer time. Macular edema is often under diagnosed, hence in patients with neuropathy and disc edema, fundus fluorescein angiography can pick up macular edema earlier which improves well with the treatment [7]. Intravitreal bevacizumab is not currently recommended as there is no promising data that supports long term resolution of disc edema following intravitreal injection. Kim et al. demonstrated short term reduction of disc edema following intravitreal Bevacizumab, however it recurred after 50 days. He found normal vitreous VEGF levels and concluded reduction of systemic VEGF is more important for resolution of disc edema rather than intravitreal VEGF levels [8].

With adequate systemic treatment, the peripapillary retinal thickness significantly and linearly correlated with the decrease in serum level of VEGF [8].

In terms of follow up of the disease activity, VEGF levels can be an imperfect marker since there could be a discordance between disease activity and response, therefore trends in VEGF levels rather than absolute values are more helpful. Similar to our patient, though there was moderate reduction of VEGF levels (249) after treatment (baseline 512 pg/ml), disc edema and peripheral leakage persisted which indicates active ongoing disease process versus progression of disease or possible treatment failure.

## Data Availability

Not applicable.

## Abbreviations

VEGF: Vascular endothelial growth factor
POEMS: Polyneuropathy, organomegaly, endocrinopathy, myeloma protein, skin changes
MRI: Magnetic resonance imaging
OCT: Optical coherence tomography
RNFL: Retinal nerve fiber layer
GCL: Ganglion cell layer
Ig: Immunoglobulin
CSF: Cerebrospinal fluid
PET: Positron emission tomography
ICP: Intracranial pressure
